# Incidence Rates and Diagnostic Trends of Perioperative Acute Transverse Myelitis in Patients Who Underwent Surgery for Degenerative Spinal Diseases: A Nationwide Epidemiologic Study of 201,769 Patients

**DOI:** 10.3390/diagnostics16010015

**Published:** 2025-12-19

**Authors:** Jihye Kim, Tae-Hwan Kim

**Affiliations:** 1 Division of Infection, Department of Pediatrics, Kangdong Sacred Heart Hospital, College of Medicine, Hallym University, Seoul 05355, Republic of Korea; jihyewiz17@gmail.com; 2 Spine Center, Department of Orthopedics, Hallym University Sacred Heart Hospital, College of Medicine, Hallym University, Anyang 14068, Republic of Korea

**Keywords:** transverse myelitis, epidemiology, degenerative, spine, disease, decompression, surgery

## Abstract

**Background**: Acute transverse myelitis (ATM) can closely mimic degenerative spinal disorders, often leading to diagnostic delay or inappropriate surgical decisions. However, its epidemiologic characteristics among patients undergoing spinal surgery remain unknown. This nationwide, population-based study investigated the incidence, perioperative diagnostic trends, and risk factors of ATM in patients treated surgically for degenerative spinal disease. **Methods**: Data were extracted from the Korean Health Insurance Review and Assessment Service database (2014–2018). Adults (>19 years) who underwent surgery for degenerative spinal disease were identified, and those with malignancy, infection, fracture, or prior myelitis were excluded. The two-year perioperative observation period (−360 to +360 days) was divided into 24 consecutive 30-day intervals. Patients were classified by ATM occurrence, and multivariable logistic regression with bootstrap validation was used to identify independent risk factors. Incidence rates were expressed per 100,000 person-years. **Results**: Among 201,769 eligible patients, 269 (0.13%) developed ATM, yielding an incidence of 67 (95% CI: 59–75) per 100,000 person-years—substantially higher than in the general population. Younger age, male sex, myocardial infarction, cerebrovascular disease, rheumatologic disease, and cervical or thoracic spinal lesions were independent predictors. Notably, 28.3% of ATM cases were diagnosed within 30 days before surgery, and 50.9% within the four-month window from three months preoperatively to one month postoperatively, indicating a marked temporal clustering around surgery. **Conclusions**: ATM occurred far more frequently among patients undergoing surgery for degenerative spinal disease than in the general population, with diagnoses peaking immediately before surgery. This pattern likely reflects diagnostic delay rather than true perioperative onset. Because ATM can clinically and radiologically resemble degenerative myelopathy, clinicians should maintain a high index of suspicion in patients presenting with atypical or rapidly progressive neurological deterioration. Early recognition may prevent unnecessary surgery and improve neurological outcomes.

## 1. Introduction

Acute transverse myelitis (ATM) is an inflammatory disorder of the spinal cord characterized by motor, sensory, and autonomic dysfunctions corresponding to the level of cord involvement [1,2,3]. Although the exact pathophysiologic mechanisms remain unclear, proposed etiologies include parainfectious, paraneoplastic, drug- or toxin-induced, systemic autoimmune, and acquired demyelinating diseases such as multiple sclerosis and neuromyelitis optica [1,2,3].

Clinically, ATM is frequently misdiagnosed or diagnosed belatedly because its presentation overlaps with a wide spectrum of neurologic disorders. In severe cases, ATM causes rapid-onset paraparesis or quadriparesis that can resemble ascending polyneuropathies such as Guillain–Barré syndrome [1,4]. An even more challenging scenario arises when ATM develops in patients who already have degenerative spinal disease with canal stenosis near the level of inflammation. Typical features—bilateral motor and sensory deficits, Lhermitte’s sign, and neuropathic axial or radicular pain—closely mimic cervical or thoracic myelopathy and radiculopathy [1,2,3]. Consequently, ATM in this context is often mistaken for mechanical spinal cord or nerve-root compression.

While degenerative spinal disease is common in the general population, ATM is rare [1,5]. Therefore, the coincidental occurrence of ATM in patients with degenerative disease is far more likely than the reverse situation. For example, a patient with cervical myelopathy evident on MRI who develops acute neurological worsening may be presumed to have disease progression requiring surgery, even if the deterioration actually results from ATM. This diagnostic pitfall may lead to inappropriate or premature surgical intervention.

To address this clinical dilemma, we conducted a nationwide epidemiologic study using a comprehensive population-based database. The study had two main objectives: (1) to identify factors associated with the occurrence of ATM in patients undergoing surgery for degenerative spinal disease and (2) to analyze perioperative incidence patterns and diagnostic timing of ATM using precisely defined time intervals.

## 2. Materials and Methods

### 2.1. Database

Data were obtained from the Korean Health Insurance Review and Assessment Service (HIRA) database, which includes healthcare claims data from all hospitals and clinics in South Korea. Diagnoses are coded according to the seventh revision of the Korean Classification of Diseases, which aligns with ICD-10, and medications are coded using the HIRA general name system based on ATC codes. This study was approved by the Institutional Review Board of our hospital (IRB No. 2020-03-009-001).

### 2.2. Study Population

**Figure 1:**
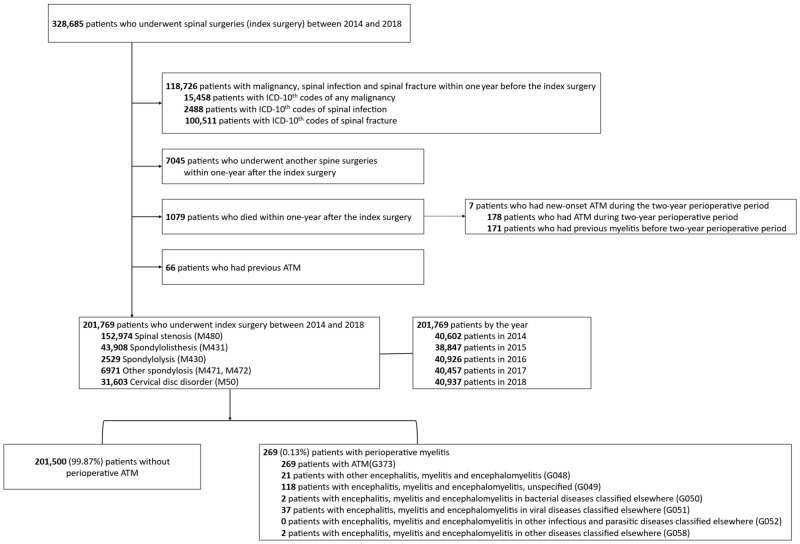
Patient enrollment. ATM; acute transverse myelitis.

We included adults (>19 years) who underwent surgical treatment for degenerative spinal disease (defined as the index surgery) between 1 January 2014 and 31 December 2018 (Figure 1), a time frame chosen to minimize confounding effects related to the COVID-19 pandemic.

Degenerative conditions were identified using ICD-10 codes for spinal stenosis (M48.0), spondylolisthesis (M43.1), spondylolysis (M43.0), spondylosis (M47.1–M47.2), and cervical disk disorder (M50) [6]. Patients with malignancy, spinal infection, or fracture within 1 year before surgery were excluded, as were those who had additional spine surgeries or died within 1 year postoperatively. To avoid including recurrent cases, patients with any prior myelitis before the perioperative observation period were also excluded.

**Figure A1:**
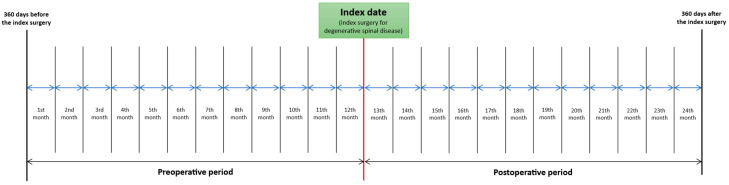
Definition of the two-year perioperative period (index period, 720 days) consisting of 24 time-intervals. Each of the 24 time-intervals is a 30-day period.

Because outcomes were ascertained within a fixed two-year perioperative window (−360 to +360 days around the index surgery, Figure A1), we restricted the analysis to incident cases by excluding any patient with a diagnostic code for myelitis recorded before the start of this window (i.e., prior to day −360). Although this exclusion minimized inclusion of recurrent or long-standing cases, it may have introduced selection bias; therefore, we precisely quantified the number of excluded patients to ensure transparency and to assess the potential impact of this exclusion on our results.

### 2.3. Identification of ATM

To minimize miscoding bias, patients with myelitis were initially identified using all relevant ICD-10 codes: acute transverse myelitis (G37.3); other encephalitis, myelitis, and encephalomyelitis (G04.8); encephalitis, myelitis, and encephalomyelitis, unspecified (G04.9); encephalitis, myelitis, and encephalomyelitis in bacterial diseases classified elsewhere (G05.0); in viral diseases classified elsewhere (G05.1); in other infectious and parasitic diseases classified elsewhere (G05.2); and in other diseases classified elsewhere (G05.8).

To confirm that the identified diagnostic codes reflected true clinical events rather than provisional or miscoded entries, we included only patients who had received corticosteroid treatment corresponding to these diagnoses, as verified using relevant drug codes (Table A1). We also evaluated the prevalence of demyelinating diseases commonly associated with ATM, including multiple sclerosis (G35) and neuromyelitis optica (G36.0).

### 2.4. Definition of Perioperative Period

A two-year perioperative observation window (720 days) was defined, comprising 24 consecutive 30-day intervals (Figure A1). This interval-based structure was designed to enable a fine-grained temporal analysis of incidence rates of acute transverse myelitis relative to the index surgery.

In this study, the term “diagnostic trend” refers to the pattern of variation in interval-specific incidence rates of acute transverse myelitis across these 24 perioperative intervals, including relative increases or decreases over time. Importantly, this term does not imply changes in the true biological incidence of the disease, but rather reflects the timing of recorded diagnoses within the claims database in relation to surgery.

### 2.5. Variables and Comorbidities

Demographic characteristics, comorbidities, and surgical information were obtained according to previously validated HIRA-based methodologies [6]. Comorbidities recorded within 1 year pre-surgery were identified by ICD-10 codes and summarized using the Charlson Comorbidity Index (CCI) [7]. Information regarding surgical procedures was obtained using electronic data interchange (EDI) codes [6]. Surgical procedures were categorized into decompressive and fusion surgeries, and for each category, the corresponding spinal region (cervical, thoracic, or lumbar) was identified (Table A2).

### 2.6. Statistical Analysis

Patients were divided into two groups according to the occurrence of ATM during the two-year perioperative period. Baseline characteristics were compared using the standardized mean difference (SMD, Cohen’s d) [8] rather than *p*-values, to quantify the magnitude of intergroup differences. This approach was adopted to emphasize the clinical relevance of observed differences, as *p*-values in large administrative datasets can be unduly influenced by sample size rather than true effect size. Variables with SMD greater than 0.1 were included in the multivariable logistic regression model to identify independent factors associated with ATM.

To assess the internal validity and robustness of the estimated coefficients, a nonparametric bootstrap resampling procedure was performed. A total of 1000 bootstrap samples were generated from the original dataset by random sampling with replacement. For each resampled dataset, the multivariable logistic regression model was re-estimated, and the resulting regression coefficients were averaged to obtain bootstrap-based estimates $\left({\bar{\beta }}_{\mathrm{boot}}\right)$. These were then compared with the corresponding coefficients from the original model (${\bar{\beta }}_{\mathrm{model}}$), and the relative bias was calculated using the following formula:

$$\mathrm{Relative}\;\mathrm{Bias}=\frac{{\bar{\beta }}_{\mathrm{boot}}-{\bar{\beta }}_{\mathrm{model}}}{{\bar{\beta }}_{\mathrm{model}}}\times 100\%$$

A relative bias value near zero indicated high internal consistency and model stability, whereas larger deviations suggested potential overfitting or sampling variability. This process enabled evaluation of the reliability of the odds ratios derived from the multivariable analysis.

Perioperative incidence rates of ATM were expressed as cases per 100,000 person-years, stratified by available clinical profiles. Specifically, the observed probability of ATM occurrence during each observation period was converted into an annualized rate, representing the number of cases expected per 100,000 individuals over one year, by dividing the number of incident cases by the total accumulated person-time and multiplying by 100,000. This standardized metric was used to enable uniform comparison of incidence rates across the 24 perioperative intervals and across different risk strata, allowing assessment of relative increases or decreases in incidence with respect to the overall cohort and specific clinical subgroups. Diagnostic trends were plotted across the 24 perioperative time intervals. All data extraction and statistical analyses were conducted using SAS Enterprise Guide version 6.1 (SAS Institute, Cary, NC, USA).

## 3. Results

### 3.1. Study Population

Of the 328,605 patients who underwent spinal surgery for degenerative spinal disease between 2014 and 2018, 118,726 were excluded due to malignancy, infection, or fracture (Figure 1). Additional exclusions included patients who underwent repeat spinal surgery or died within one year after the index surgery (7045 and 1079 patients, respectively), as well as those with a prior diagnosis of ATM (*n* = 66).

Among the 1079 patients who died within one year after the index surgery, 7 had a new-onset ATM. In addition, among the 66 excluded patients with a previous history of ATM, 2 also developed a new-onset ATM during the observation period. As a result, 9 patients who experienced a new-onset ATM within the index period were excluded from the final cohort. These exclusions may have introduced a minor selection bias but were necessary to ensure that only patients with complete perioperative follow-up and without recurrent cases were analyzed.

After applying all exclusion criteria, a total of 201,769 patients were included in the final analysis, among whom 269 (0.13%) developed ATM during the two-year perioperative period.

### 3.2. Diagnostic Confirmation and Annual Incidence

All 269 cases carried the ICD-10 code G37.3 for ATM (Figure 1). The next most frequent accompanying codes were unspecified encephalomyelitis (G04.9, *n* = 118) and viral encephalomyelitis (G05.1, *n* = 37). None had multiple sclerosis or neuromyelitis optica. All patients received systemic corticosteroids (median duration = 21 days). The overall incidence of ATM was 67 (95% CI: 59–75) per 100,000 person-years, demonstrating a gradual increase across the study period (Table 1).

### 3.3. Patient Characteristics

ATM occurred more frequently in younger male patients treated at tertiary hospitals (Table 2).

Comorbidities associated with ATM included myocardial infarction (SMD = 0.516), hemiplegia/paraplegia (0.230), Parkinson’s disease (0.205), cerebrovascular disease (0.181), dementia (0.164), and rheumatologic disease (0.139) (Table 3).

Conversely, renal disease, osteoporosis, heart failure, peptic ulcer disease, and uncomplicated diabetes were more common among patients without ATM. CCI scores did not differ significantly between groups. ATM was predominantly associated with cervical (SMD = 0.883) and thoracic (SMD = 0.829) lesions, and rarely with lumbar pathology (Table 4).

Taken together, these findings summarize the baseline characteristics of patients with ATM, and the corresponding perioperative incidence rates according to each clinical profile are presented in Table 2, Table 3 and Table 4.

### 3.4. Factors Associated with Occurrence of ATM: Multivariable Analysis

Independent predictors of ATM included younger age, male sex, myocardial infarction, cerebrovascular disease, rheumatologic disease, and cervical or thoracic surgical level (Table A3).

**Figure 2:**
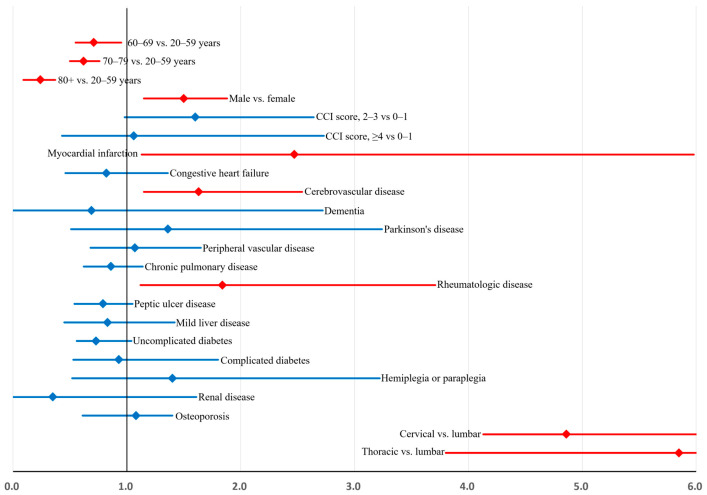
Factors associated with acute transverse myelitis. The *x*-axis represents bootstrap-adjusted odds ratios.

The corresponding bootstrap-adjusted odds ratios with their 95% confidence intervals are illustrated in Figure 2.

Bootstrap-adjusted ORs exhibited minimal relative bias (−6.4 to 8.5%).

### 3.5. Perioperative Diagnostic Trend of ATM

**Figure 3:**
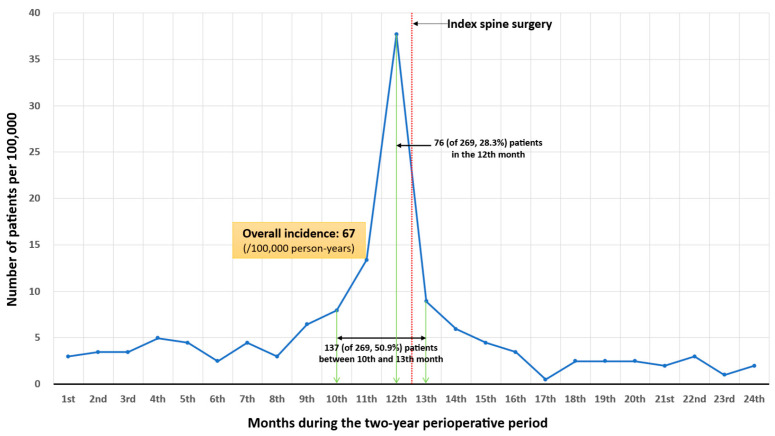
The overall diagnostic trend of acute transverse myelitis. The *x*-axis represents 24 time-intervals of the two-year perioperative period.

Overall diagnostic patterns revealed a steep rise in ATM diagnoses shortly before surgery (Figure 3).

**Figure 4:**
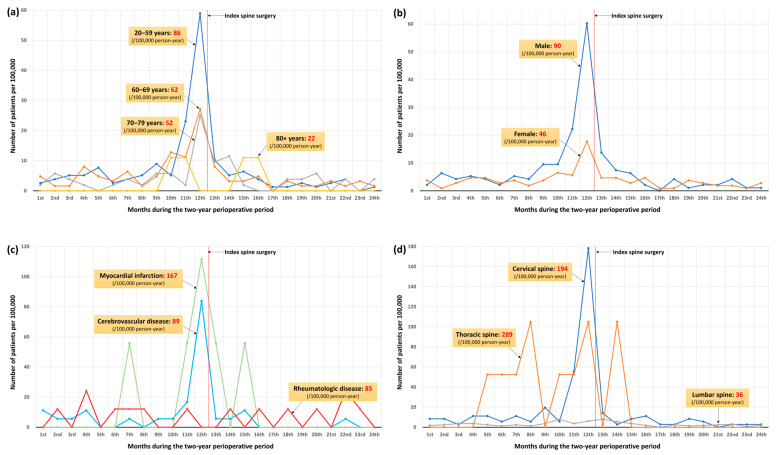
Diagnostic trends according to factors associated with acute transverse myelitis: (**a**) by age groups; (**b**) by sex; (**c**) by comorbidities, and (**d**) by spinal regions. The *x*-axis represents 24 time-intervals of the two-year perioperative period.

Specifically, 28.3% of cases were identified within 30 days preoperatively, and 50.9% between 3 months before and 1 month after surgery. Subgroup trends by associated factors (age, sex, comorbidities, spinal region) (Figure 4) generally paralleled the overall pattern, except in patients with rheumatologic disease or thoracic myelopathy, whose diagnoses were more evenly distributed throughout the observation period.

## 4. Discussion

This nationwide, population-based study is the first to elucidate the epidemiology of ATM among patients undergoing surgery for degenerative spinal disease. We identified 269 cases of ATM, corresponding to an incidence of 67 per 100,000 person-years—substantially higher than rates reported in the general population [1,5]. Younger male patients and those with myocardial infarction, cerebrovascular, or rheumatologic diseases, particularly involving the cervical or thoracic spine, were at increased risk. Notably, approximately half of all ATM cases (50.9%) were diagnosed within a narrow four-month window spanning from three months before to one month after the index surgery, indicating a marked concentration of diagnoses within this perioperative timeframe. This diagnostic pattern was consistently observed across most risk factor subgroups, including age, sex, and major comorbidities, but was less evident in patients with thoracic spinal lesions or rheumatologic disease.

The reported annual incidence of ATM in the general population ranges from 1.3 to 24.6 per million [1,5], whereas rates are substantially higher in autoimmune cohorts—up to 1–3% in systemic lupus erythematosus [9,10] and approximately 1% in Sjögren’s syndrome [11]. In our surgical cohort, the observed incidence of ATM was considerably higher than that reported in the general population and approached the levels seen in autoimmune disorders. However, this finding should be interpreted with caution, as differences in observation periods and follow-up completeness among studies, together with the increasing accessibility and utilization of spinal MRI, may have led to potential over-diagnosis of ATM rather than a true rise in its occurrence (Table 1). In our cohort, the incidence of ATM showed a marked decline in older patients, particularly those aged over 80 years (Figure 4a), which was consistent with the age-related pattern typically observed in the general population with ATM [1,10].

The most important finding of this study is not merely the higher incidence of ATM among patients with degenerative spinal disease, but rather the diagnostic timing, which was markedly concentrated in the period preceding surgery. The observed preoperative increase in ATM incidence can be interpreted in two ways: it may represent either a true rise in disease occurrence among patients with degenerative spinal disease or a delay in diagnosis. Considering the overall temporal pattern and clinical plausibility, we believe that delayed diagnosis is the more likely explanation. ATM is often difficult to distinguish from cervical or thoracic myelopathy caused by progression of degenerative disease. In patients who have already undergone MRI confirming degenerative myelopathy, a subsequent worsening of neurological deficits is typically interpreted by clinicians as aggravation of the pre-existing condition. As a result, they are more likely to proceed with active treatment for degenerative disease rather than performing additional imaging studies to rule out ATM [12], which inevitably leads to diagnostic delay. Moreover, the elevated incidence of ATM did not return to baseline immediately after surgery but remained higher for up to one month postoperatively. The fact that ATM showed a persistently high incidence during the early postoperative period suggests that a portion of these cases may represent patients whose new-onset ATM had initially been misdiagnosed as progression of degenerative myelopathy and who may have undergone surgery while the condition was unrecognized as ATM.

Interestingly, the 76 patients who were diagnosed with ATM in the 12th month—immediately before the index surgery—underwent spinal surgery despite having already been diagnosed with ATM preoperatively. At first glance, this may appear to represent premature surgical intervention, considering that the clinical nadir of ATM typically develops within 4 h to 21 days [13]. However, we interpret this finding differently. Although, in theory, the clinical presentation of ATM—characterized by rapidly progressive neurological deficits not confined to a specific lesion level and by MRI findings of central T2-hyperintense lesions extending over two or more segments [12]—should be readily distinguishable from degenerative myelopathy, in actual clinical practice this distinction is often far from straightforward. For instance, ATM can occasionally present atypically with a focal lesion and slow progression, whereas degenerative myelopathy may acutely worsen when accompanied by a traumatic event. Consequently, in situations where the diagnosis is ambiguous, spine surgeons may proceed with decompressive surgery out of concern for missing the optimal therapeutic window—a decision that is likely to occur more frequently when such atypical ATM coincides with a traumatic event.

Considering these diagnostic and treatment challenges, the present study is the first to report the incidence of ATM in patients with degenerative spinal disease. The incidence of ATM was 67 per 100,000 person-years, which may appear rare at first glance. However, this rate is substantially higher than that in the general population, and clinicians with sufficient experience in degenerative spinal disease will likely have encountered, at least once, a patient with concurrent ATM—either directly or indirectly. When clinicians first face an unexpected case of concomitant ATM, diagnostic delay or unnecessary surgery can easily occur. Even for those who have previously managed such cases, determining the optimal management strategy remains difficult, and an inappropriate decision can lead to devastating neurological outcomes. Against this background, our study utilized a nationwide population-based database to move beyond simply estimating incidence. We analyzed perioperative temporal patterns and risk factors for ATM occurrence in this population, providing baseline incidence data stratified by readily available clinical characteristics and supported by bootstrap validation of statistical robustness—the largest dataset reported to date (*n* = 201,769). Furthermore, by comparing the timing of surgical treatment for degenerative spinal disease with the diagnostic timing of ATM, we quantified the extent of diagnostic delay in real-world practice and offered insights into how surgeons make operative decisions when ATM is concomitantly present.

Nevertheless, several important limitations inherent to claims database–based research should be acknowledged. First, potential discrepancies between clinical reality and administrative coding may introduce misclassification bias. ATM is fundamentally a radiological and clinical diagnosis; however, the claims database does not include spinal MRI findings, cerebrospinal fluid data, or detailed neurological examination results. Consequently, diagnostic misclassification cannot be completely excluded, despite our efforts to improve specificity by combining ICD diagnostic codes with corticosteroid treatment records. Second, the database lacks information on the anatomical severity of degenerative spinal disease, including the degree of spinal stenosis, extent of spinal cord compression, and detailed imaging characteristics. In addition, data on traumatic events, acute neurological deterioration, or surgical urgency are unavailable, precluding adjustment for anatomical severity or trauma-related triggers that may influence disease onset or progression. Third, neurological recovery, functional outcomes, and surgical results could not be assessed, as postoperative clinical outcome measures are not captured in the claims data. Therefore, the incidence and diagnostic trends observed in this study should not be interpreted as evidence to guide clinical treatment decisions, and the relationship between diagnosis, intervention, and neurological prognosis remains beyond the scope of the present analysis. Future studies integrating administrative databases with detailed imaging, neurological assessments, and longitudinal outcome data are warranted to address these limitations and to establish clinically actionable diagnostic and therapeutic strategies.

This nationwide, population-based study is the first to characterize the epidemiology, diagnostic patterns, and risk factors of ATM among patients undergoing surgery for degenerative spinal disease. The incidence of ATM was 67 per 100,000 person-years, markedly higher than that in the general population. Notably, nearly half of the cases were diagnosed within the four-month window spanning from three months before to one month after surgery. ATM can closely mimic degenerative cervical or thoracic myelopathy, making early recognition challenging, particularly in patients with pre-existing spinal cord compression or concurrent traumatic events. These findings highlight the importance of maintaining a high index of suspicion for ATM in patients with atypical or rapidly progressive neurological deterioration despite imaging evidence of degenerative disease. Awareness of this diagnostic pitfall may help clinicians avoid unnecessary surgery, ensure timely evaluation, and ultimately improve neurological outcomes in this rare but clinically significant condition.

## Figures and Tables

**Table 1:** Annual incidence of acute transverse myelitis.

Year	Spinal Surgery (*n*)	(*n*)	Incidence (per 100,000 Person-Year)	95% CI
2014	40,602	40	49	[28–71]
2015	38,847	37	48	[32–63]
2016	40,926	48	59	[42–75]
2017	40,457	61	75	[56–94]
2018	40,937	83	101	[80–123]
All	201,769	269	67	[59–75]

**Table 2:** Comparison of demographics.

Variables	Categories	All	Patients Without Acute Transverse Myelitis (*n* = 201,500)	Patients with Acute Transverse Myelitis (*n* = 269)	Standardized Mean Difference	Incidence Rates (per 100,000 Person-Year)
Age	Mean ± SD	62.1 ± 12.0	62.2 ± 12.0	59.1 ± 12.2	0.258	
	20–59	78,049	77,915 (38.7)	134 (49.8)		86
	60–69	62,516	62,439 (31)	77 (28.6)		62
	70–79	52,116	52,062 (25.8)	54 (20.1)		52
	80+	9088	9084 (4.5)	4 (1.5)		22
Sex	Male	94,528	94,357 (46.8)	171 (63.6)	0.377	90
	Female	107,241	107,143 (53.2)	98 (36.4)		46
Region	Urban	167,931	167,702 (83.2)	229 (85.1)	0.079	68
	Rural	33,838	33,798 (16.8)	40 (14.9)		59
Hospital	Tertiary	35,108	34,999 (17.4)	109 (40.5)	0.648	155
	General	39,997	39,931 (19.8)	66 (24.5)		83
	Others	126,664	126,570 (62.8)	94 (34.9)		37

Data are presented as mean ± standard deviation or *n* (%). Intergroup differences were estimated using the standardized mean difference (SMD), and a significant difference was defined as a standard SMD > 0.1.

**Table 3:** Comparison of comorbidities.

Variables	Categories	All	Patients Without Acute Transverse Myelitis (*n* = 201,500)	Patients with Acute Transverse Myelitis (*n* = 269)	Standardized Mean Difference	Incidence Rates (per 100,000 Person-Year)
Charlson comorbidity index score	Mean ± SD	1.14 ± 1.28	1.14 ± 1.28	1.21 ± 1.30	0.055	
	0–1	141,802	141,629 (70.3)	173 (64.3)		61
	2–3	48,414	48,333 (24)	81 (30.1)		84
	≥4	11,553	11,538 (5.7)	15 (5.6)		65
Comorbidities	Myocardial infarction	1792	1787 (0.9)	6 (2.2)	0.516	167
	Congestive heart failure	6329	6323 (3.1)	6 (2.2)	0.193	47
	Cerebrovascular disease	17,894	17,862 (8.9)	32 (11.9)	0.181	89
	Dementia	2239	2235 (1.1)	4 (1.5)	0.164	89
	Parkinson’s disease	1557	1554 (0.8)	3 (1.1)	0.205	96
	Peripheral vascular disease	21,991	21,961 (10.9)	30 (11.2)	0.014	68
	Chronic pulmonary disease	46,358	46,301 (23)	57 (21.2)	0.057	61
	Rheumatologic disease	8251	8242 (4.1)	14 (5.2)	0.139	85
	Peptic ulcer disease	34,894	34,856 (17.3)	38 (14.1)	0.132	54
	Liver disease					
	Mild	12,571	12,553 (6.2)	18 (6.7)	0.042	72
	Moderate to severe	163	163 (0.1)	0 (0)	-	0
	Diabetes					
	Uncomplicated	42,078	42,031 (20.9)	47 (17.5)	0.121	56
	Complicated	12,481	12,466 (6.2)	15 (5.6)	0.061	60
	Hemiplegia or paraplegia	1489	1486 (0.7)	3 (1.1)	0.230	101
	Renal disease	3294	3292 (1.6)	2 (0.7)	0.439	30
	End stage renal disease	571	571 (0.3)	0 (0)	-	0
	Osteoporosis	30,314	30,286 (15)	28 (10.4)	0.232	46

Data are presented as mean ± standard deviation or *n* (%). Intergroup differences were estimated using the standardized mean difference (SMD), and a significant difference was defined as a standard SMD > 0.1.

**Table 4:** Comparison of the index spine surgery.

Variables	Categories	All	Patients Without Acute Transverse Myelitis (*n* = 201,500)	Patients with Acute Transverse Myelitis (*n* = 269)	Standardized Mean Difference	Incidence Rates (per 100,000 Person-Year)
Cervical surgery	All	35,879	35,740 (17.7)	139 (51.7)	0.883	194
	Decompressive surgery	21,260	21,175 (10.5)	85 (31.6)		200
	Fusion surgery	14,619	14,565 (7.2)	54 (20.1)		185
Thoracic surgery	All	1904	1893 (0.9)	11 (4.1)	0.829	289
	Decompressive surgery	1460	1454 (0.7)	6 (2.2)		205
	Fusion surgery	444	439 (0.2)	5 (1.9)		563
Lumbar surgery	All	163,986	16,3867 (81.3)	119 (44.2)	0.939	36
	Decompressive surgery	127,364	127,272 (63.2)	92 (34.2)		36
	Fusion surgery	36,622	36,595 (18.2)	27 (10)		37

## Data Availability

The data used in this study were obtained from the Health Insurance Review and Assessment Service (HIRA), a government-managed national healthcare database of the Republic of Korea. Due to legal and ethical restrictions imposed by the data provider, the raw dataset cannot be publicly shared or exported. However, the tables generated and the analytical results supporting the findings of this study are available from the corresponding author upon reasonable request.

## Appendix A

**Table A1:** HIRA general name codes for used steroids.

Category of Steroid	Type of Steroid	ATC Code	HIRA General Name Code
Oral steroid	deflazacort 6 mg	H02AB13	140801ATB
	dexamethasone 0.5 mg	H02AB02	141901ATB
	dexamethasone 0.75 mg	H02AB02	141903ATB
	betamethasone 0.25 mg + d-chlorpheniramine 2 mg	H02AB01	296900ATB
	hydrocortisone 10 mg	H02AB09	116401ATB
	hydrocortisone 5 mg	H02AB09	170901ATB
	methylprednisolone 4 mg	H02AB04	193302ATB
	methylprednisolone 1 mg	H02AB04	193305ATB
	prednisolone 5 mg	H02AB06	217001ATB
	triamcinolone 1 mg	H02AB08	243201ATB
	triamcinolone 2 mg	H02AB08	243202ATB
	triamcinolone 4 mg	H02AB08	243203ATB
	fludrocortisone 100 μg	H02AA02	160201ATB
Intravenous steroid	dexamethasone 4 mg	H02AB02	142030BIJ
	dexamethasone 4 mg	H02AB02	142230BIJ
	dexamethasone 5 mg	H02AB02	142232BIJ
	betamethasone 4 mg	H02AB01	116530BIJ
	hydrocortisone 100 mg	H02AB09	171201BIJ
	methylprednisolone 125 mg	H02AB04	193601BIJ
	methylprednisolone 40 mg	H02AB04	193603BIJ
	methylprednisolone 500 mg	H02AB04	193604BIJ
	triamcinolone 10 mg	H02AB08	243336BIJ
	triamcinolone 40 mg	H02AB08	243335BIJ
	triamcinolone 40 mg	H02AB08	243337BIJ

**Table A2:** HIRA therapeutic codes for spinal surgery.

Surgical Methods	Spinal Regions	HIRA Therapeutic Codes
Decompressive surgery	Cervical	N2491, N2492, N0491, N1491, N1497, N2497
	Thoracic	N1492, N1498, N2498
	Lumbar	N0492, N1493, N1499, N2499
Fusion surgery	Cervical	N2461, N0464, N2463, N2467, N2468, N0467, N2469
	Thoracic	N0465, N2464, N2465, N2466, N0468
	Lumbar	N0466, N1466, N0469, N1460, N1469, N2470

**Table A3:** Factors associated with acute transverse myelitis.

Variables	Categories	Model 1 (Fully Adjusted)		Model 2 (Bootstrap Validation After Fully Adjusted)	
		Adjusted Odds Ratio (95% Confidence Interval)	*p*-Value	Adjusted Odds Ratio (95% Confidence Interval)	Relative Bias (%)
Age	60–69 vs. 20–59	0.75 [0.56–1.00]	0.051	0.71 [0.55–0.95]	15.75
	70–79 vs. 20–59	0.63 [0.44–0.88]	0.007	0.62 [0.50–0.76]	1.25
	80+ vs. 20–59	0.26 [0.09–0.70]	0.008	0.24 [0.09–0.37]	5.97
Sex	Male vs. female	1.45 [1.11–1.90]	0.006	1.50 [1.15–1.88]	8.48
Charlson comorbidity index score	2–3 vs. 0–1	1.58 [0.78–1.20]	0.086	1.60 [0.98–2.64]	2.54
	≥4 vs. 0–1	1.14 [0.37–3.53]	0.822	1.06 [0.43–2.73]	−53.13
Comorbidities	Myocardial infarction	2.33 [1.19–5.81]	0.036	2.47 [1.13–5.98]	6.87
	Congestive heart failure	0.77 [0.33–1.80]	0.547	0.82 [0.46–1.36]	−23.66
	Cerebrovascular disease	1.65 [1.13–2.42]	0.010	1.63 [1.15–2.54]	−2.58
	Dementia	1.54 [0.55–4.30]	0.414	0.69 [0–2.72]	−187.75
	Parkinson’s disease	1.70 [0.54–5.35]	0.368	1.36 [0.51–3.24]	−41.13
	Peripheral vascular disease	1.07 [0.69–1.66]	0.770	1.07 [0.68–1.65]	−0.13
	Chronic pulmonary disease	0.92 [0.64–1.31]	0.636	0.86 [0.62–1.14]	71.78
	Rheumatologic disease	1.90 [1.11–3.84]	0.042	1.84 [1.12–3.71]	−5.27
	Peptic ulcer disease	0.76 [0.51–1.14]	0.186	0.79 [0.54–1.05]	−11.09
	Liver disease				
	Mild	0.92 [0.54–1.55]	0.744	0.83 [0.45–1.42]	108.25
	Moderate to severe	-	-		
	Diabetes				
	Uncomplicated	0.77 [0.33–1.80]	0.192	0.73 [0.56–1.04]	23.74
	Complicated	0.94 [0.46–1.94]	0.875	0.93 [0.53–1.80]	27.85
	Hemiplegia or paraplegia	1.74 [0.55–5.48]	0.346	1.40 [0.52–3.22]	−39.24
	Renal disease	0.53 [0.12–2.31]	0.401	0.35 [0–1.61]	68.51
	End stage renal disease	-	-		
	Osteoporosis	1.06 [0.70–1.63]	0.778	1.08 [0.61–1.40]	24.82
Region	Cervical vs. lumbar	4.75 [3.63–6.21]	<0.001	4.86 [4.13–6.14]	1.45
	Thoracic vs. lumbar	6.61 [3.53–12.36]	<0.001	5.85 [3.80–9.54]	−6.42

Adjustments were done by age groups, sex, CCI score, myocardial infarction, congestive heart failure, cerebrovascular disease, dementia, Parkinson’s disease, peripheral vascular disease, chronic pulmonary disease, rheumatologic disease, peptic ulcer disease, uncomplicated diabetes, hemiplegia or paraplegia, renal disease, osteoporosis, and spinal regions. Relative bias was estimated as the difference between the mean bootstrapped regression coefficient estimates (model 2) and the mean parameter estimates of multivariable model (model 1) divided by the mean parameter estimates of multivariable model (model 2).
